# New synchrotron powder diffraction facility for long-duration experiments

**DOI:** 10.1107/S1600576716019750

**Published:** 2017-02-01

**Authors:** Claire A. Murray, Jonathan Potter, Sarah J. Day, Annabelle R. Baker, Stephen P. Thompson, Jon Kelly, Christopher G. Morris, Sihai Yang, Chiu C. Tang

**Affiliations:** a Diamond Light Source, Harwell Campus, Didcot, Oxfordshire OX11 0DE, UK; b School of Chemistry, The University of Manchester, Oxford Road, Manchester M13 9PL, UK

**Keywords:** synchrotron powder diffraction, long-duration experiments, instrumentation

## Abstract

A new synchrotron X-ray powder diffraction instrument has been built and commissioned for long-duration experiments on beamline I11 at Diamond Light Source. The concept is unique, with design features to house multiple experiments running in parallel, in particular with specific stages for sample environments to study slow kinetic systems or processes. The instrument benefits from a high-brightness X-ray beam and a large area detector. Diffraction data from the commissioning work have shown that the objectives and criteria are met. Supported by two case studies, the results from months of measurements have demonstrated the viability of this large-scale instrument, which is the world’s first dedicated facility for long-term studies (weeks to years) using synchrotron radiation.

## Abstract 2

The world’s first dedicated synchrotron instrument for long-duration experiments has been built and commissioned at Diamond Light Source. The new beamline I11 user facility is designed for the study of slow kinetics in polycrystalline materials.

## 1. Introduction   

### 1.1. Synchrotron standard user mode   

Synchrotron beamlines and their instruments are built to harness the photon beam power of synchrotron radiation (SR), which has special properties – such as high brightness, broad spectral range (energy/wavelength tunability), polarization and time structure – ideally suited to providing detailed and accurate structural information that is difficult to obtain from conventional sources. In the main, experiments are conducted by numerous and diverse user groups drawn from a large academic and industrial community spread across a wide range of physical and life sciences. The common *modus operandi* for such facilities is that approved user proposals are allocated a short duration of beamtime, typically ranging from a few hours to a few days, in which to perform their experiment. Ideally, the experiment’s specific aim(s) should be achieved within the allocated beamtime and the results should be subsequently published. This model has been in operation very successfully for many years at all major SR laboratories worldwide and will certainly continue into the future because of the high impact and productive nature of the process. To review the huge contributions from SR experiments to the progress of science is beyond the scope of this paper, but many cases are given in synchrotron text books (*e.g.* Willmott, 2011 ▸) and in the annual reports and reviews produced by the facilities themselves, as well as the extensive body of published papers citing the use of SR in their work. With technological advances in instrumentation, detection, computing power, automation and remote access, SR facilities are developing new modes of access, designed to increase speed, efficiency and throughput of user experiments, such as on the macromolecular beamlines at Stanford Synchrotron Radiation Lightsource (Smith *et al.*, 2010 ▸) and at the Diamond Light Source (Aller *et al.*, 2015 ▸) and the mail-in service on beamline 11-BM at the Advanced Photon Source (Toby *et al.*, 2009 ▸).

### 1.2. Long-duration experiments (LDEs)   

However, as both user and sample throughput rates increase there are a class of experiments that are increasingly excluded by these developments, which nevertheless could greatly benefit from the application of SR. Many materials require long incubation periods in order to ‘cure’ or, alternatively, to show the effects of slow degradation due to ageing. Some materials under long-term exposure to non-ambient *operando* conditions undergo very slow transforming reactions, while others exhibit the results of an accumulative build-up of effects over extended periods of high-frequency duty cycles. All these processes can be subtle and take weeks to months or even years in order to either show gross manifestation or run to completion.

At present, off-line processing with before and after SR measurements is the norm, but valuable structural information on growth, change and intermediate phases is missed or indeed lost, particularly when samples are removed from host environments. The understanding of ‘slow’ kinetics is thus always limited to *ex situ* data or indirect evidence from secondary measurements such as thermal analysis. Typical research areas affected by these issues include, for example, long-term corrosion studies (*i.e.* electrochemistry, engineering, conservation), development of manufacturing/processing techniques, crystal growth, ageing at medium–low temperatures, work/duty cycle hardening (*e.g.* engineering materials, environmental processing), electrochemical cycling (*e.g.* energy storage materials, solid oxide fuel cells), interactions with various atmospheres (*e.g.* weathering, planetary surfaces, pollution effects, conservation) and simulation of geological processes (*e.g.* mineral formation, leeching, hydro­logical cycling). In some cases, insights can be gained by increasing reaction rates through the use of elevated temperatures, but kinetic outcomes can, and often are, different to thermodynamic outcomes and their value can be limited. There is therefore a clear need for a facility that allows slow processes to be studied, but which also brings to bear all of the power, resolution and refinement of SR techniques as well as exploiting the high-throughput potential that high photon rates can offer.

### 1.3. SR powder diffraction and new LDE facility   

With the increasing brightness of photon sources and advancing beamline and detector technologies, much of the developmental emphasis has been on high-throughput and fast data collection in existing synchrotron facilities. For example, an entire powder diffraction pattern can be routinely collected on a time scale of a few seconds to milliseconds using microstrip or pixel area detectors (Bergamaschi *et al.*, 2009 ▸; Thompson *et al.*, 2011 ▸). Ironically, it is this trend towards ever faster data collection and sample throughput that has allowed us both to develop the concept of and to build the world’s first synchrotron X-ray powder diffraction (SXPD) LDE facility. The new facility takes the form of an additional specially constructed end-station to the existing ultra-high-resolution and time-resolved powder diffraction beamline (I11) at the Diamond Light Source (UK). The new end-station is dedicated to hosting multiple long-term experiments, all running in parallel.

### 1.4. SXPD beamline I11   

**Figure 1:**
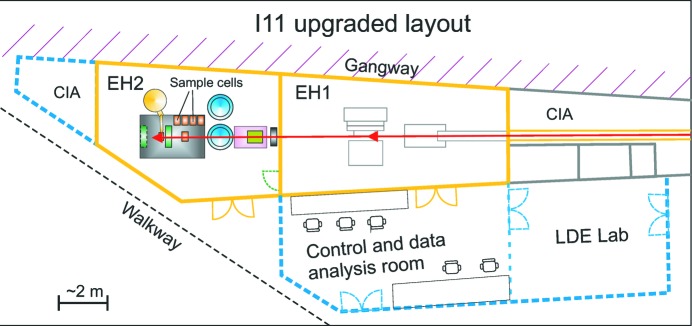
The upgraded layout of beamline I11 at Diamond (schematic).

Detailed technical descriptions of I11, its detection arrangements and its performance have been given previously (Thompson *et al.*, 2009 ▸, 2011 ▸; Parker *et al.*, 2011 ▸). However, in brief, a high-brightness X-ray beam optimized at 15 keV (λ ≃ 0.826 Å) can be delivered from the in-vacuum undulator to the beamline, which was originally a standard two-hutch design. The optics hutch hosts a double-crystal monochromator with Si(111) crystals and harmonic rejection mirrors. The experimental hutch (EH1) houses a heavy-duty diffractometer with two detection systems: 45 multi-analysing crystals (MACs) and scintillator detectors arranged over five arms for ultra-high-resolution experiments, and a position-sensitive detector comprising 18 pixelated Si strip detector modules arranged to give a 90° arc for high-resolution time-resolved studies. The two detection systems provide resolving powers of Δ*d*/*d* of ∼10^−4^–10^−6^ and ∼10^−3^–10^−4^, respectively. To accommodate the new LDE facility, a second experimental hutch (EH2) has been constructed in series with EH1 (Fig. 1 ▸). Although the available floor space was limited, a footprint of ∼40 m^2^ for EH2 was achieved. A new control room and support laboratory for EH2 was also constructed for the off-line development, commissioning and testing of LDE sample cells.

In this paper, we report on the new purpose built LDE facility, which has been designed to address the needs of a wide and diverse range of scientific investigations. Here the design concepts are described, along with the installed hardware for hosting multiple sample stages, cells and services capable of providing a range of non-ambient environments. The commissioning of a large pixelated area detector for fast data acquisition is also described, along with the data collection strategy. To demonstrate the effectiveness of this new facility, commissioning results from two contrasting science cases are presented. In the first, the slow *in situ* precipitation of the hydrated Mg sulfate mineral meridianiite from an aqueous solution of MgSO_4_ is followed. The hydrated phase is believed to be widespread on the surface of Mars (Peterson & Wang, 2006 ▸; Peterson *et al.*, 2007 ▸) and was formed inside a specifically designed low-temperature cell. In the second study, the long-term stability of the metal–organic framework material NOTT-300 was investigated. This is a potential supramol­ecular material for greenhouse gas capture (Yang, Lin *et al.*, 2012 ▸; Yang *et al.*, 2015 ▸). However, to assess its potential as an applied material, its structural longevity with captured toxic gas needs to be investigated.

## 2. I11 LDE facility: philosophy and design   

### 2.1. Mode of use, criteria and key specifications   

Unlike normal SR experiments, which require the continual collection of data on a scale of seconds to minutes to hours within an allocated beamtime slot, an LDE, where noticeable changes may take weeks to develop, does not need continual measurement but rather regular monitoring (*e.g.* weekly measurements) over an extended period lasting weeks to months to years. However, tying up a whole beamline with a single experiment for such a long period is simply not viable in terms of access for other users, allocation of resources and scientific risk: or indeed, from a funding agency point of view, value for money. The operational imperative is therefore to run an optimum number of multiple experiments, each receiving a scheduled time-slice access to the beam. In order to realize this, the use of the available floor space was optimized to accommodate the maximum number of possible experiments and to employ fast detection methods to ensure that each experiment has sufficient exposure to the X-ray beam to return good quality data. In addition, the design criteria of automated operation for quick changeovers between the different experiments must be included in order to make the most efficient use of the available LDE beamtime slot. This is due to the added LDE facility on I11 being built in series with EH1 which, with its highly active user schedule, is both in high demand and highly productive. LDE operation therefore needs to be as unobtrusive to normal operations as possible.

The use of an area detector, transmission geometry and high-energy X-ray beam allows us to meet the fast detection criterion as whole Debye–Scherrer (powder) rings can be captured on a timescale of seconds and integrated to give two-dimensional diffraction patterns of good statistical quality. Although the resolution (Δ*d*/*d* ≃ 10^−3^–10^−4^) is lower than can be achieved using slower angle dispersive or angle scanning measurements, which only capture a very small portion of the Debye–Scherrer rings, it should be sufficient for most *in situ* cycling and processing experiments since the primary objective in most LDEs is phase development, for which ultra-high resolution is not generally required. The I11 LDE design criteria can be summarized as follows:

(1) Multiple experiments, with cells running in parallel

(2) Transmission powder diffraction (Δ*d*/*d* ≃ 10^−3^–10^−4^) using a fast area detector

(3) Automation features for quick changeover between LDEs, data collection and reduction

(4) Standardized mounting and service interfaces for uniformity and ease of implementation

(5) Continuous monitoring of parametric data (*e.g*. cell temperature, pressure, humidity *etc*.)

(6) High beam brightness at sufficiently high energy for penetration and fast data acquisition

The existing I11 X-ray source and beamline optical elements are capable of delivering a high-brightness monochromatic beam up to 30 keV such that the combination of beam energy, brightness and area detector make it possible to collect a complete dataset in EH2 in a matter of minutes. The main components are listed in Table 1 ▸, together with the specifications for the detection system, acquisition speed and beam size for LDEs.

### 2.2. LDE hutch layout   

**Figure 2:**
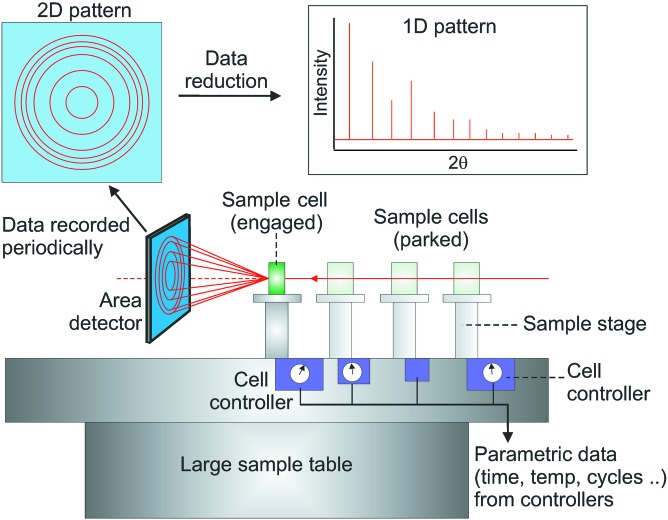
Schematic representation of the diffraction geometry and design concept.

A schematic of the EH2 LDE concept is shown in Fig. 2 ▸. The sample cells for each LDE are mounted on motorized linear stages and at the start of a data collection run are initially at parked positions away from the X-ray beam. The detector is also motorized and is located in a large frame that is attached to, and can move along, the sample table, giving it travel along the beam. The sequence of operations is for the first cell to move across the sample table into the beam, after which the detector moves to the necessary predetermined distance required to view the cell. Data collection is then triggered, after which the cell returns to its parked position and the detector retreats. This process is then sequentially repeated for all active cells along the sample table. In this way many measurements for multiple LDEs can be collected within a fixed beamtime allocation. The measurements themselves are made on a weekly basis during the operational beam cycle to produce long-time-span datasets for each LDE. During shutdowns, cell cycling can either continue or be suspended for the duration, with diffraction data collection resuming following machine start up.

In order to produce a design that maximizes the use of the available space in EH2, five generic types of experiment stage were identified that would be capable of accommodating as many different experiments as possible:

(1) Small cells: with limited services (electricity and sensor connections), for small-scale environments requiring electrical services for monitoring or control only

(2) Medium cells: with electrical, sensor and gas/fluid services, for cells with heating, cooling or liquid circulation

(3) Large cells: again with electrical and fluid services, to house experiments aimed at simulating industrial conditions

(4) Type-R: a robotic sample changer and a small dedicated linear stage, for static ageing experiments with no service requirements

(5) Type-T: a special sample table (services possible) with breadboard top, for those experiments which do not easily fit on any of the above, or with bespoke requirements

**Figure 3:**
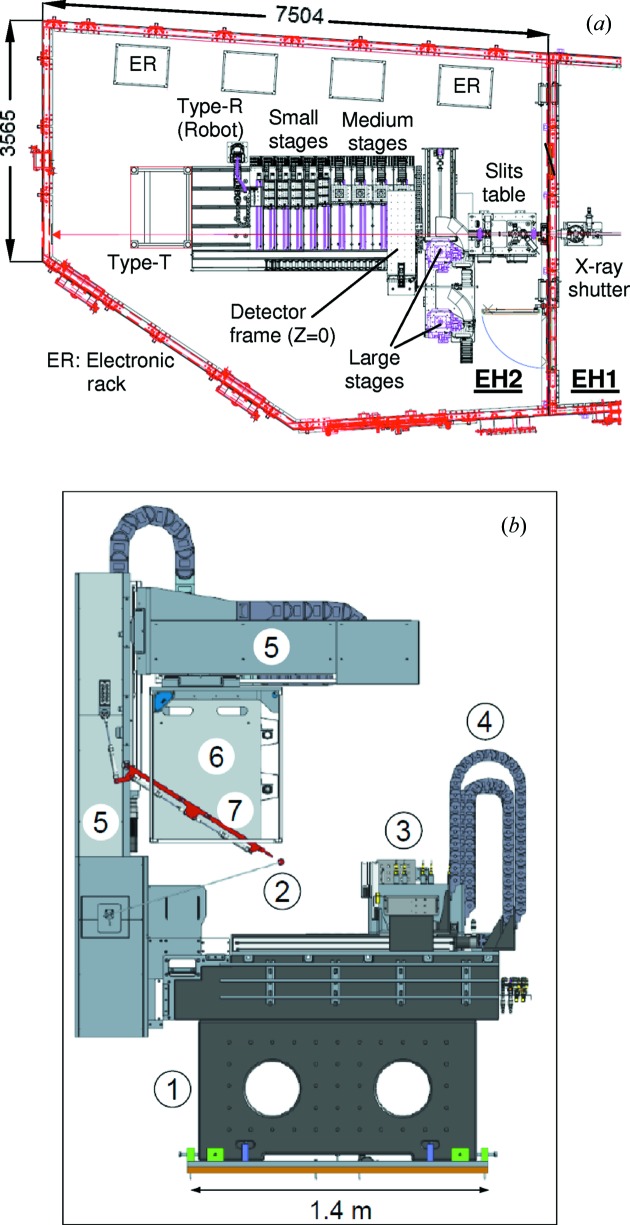
(*a*) Drawing of the LDE hutch layout (all dimensions are in millimetres) with the key components indicated. (*b*) A CAD drawing of the experimental area (viewed along the X-ray beam). The key components are labelled as (1) large granite table, (2) tungsten beam stop, (3) small and medium stages, (4) energy (electrical) chains, (5) large *XYZ* detector frame, (6) detector bracket and (7) retractable arm with lead disc.

The technical specifications for each cell type are listed in Table 2 ▸. The first three stage types are grouped according to the size and weight of the possible sample cells that could be housed on them. These are summarized in Table 3 ▸. Apart from the Type-T, all stages are equipped with high-precision motors and encoders. The stages in each type are identical in their range of motion and loading capacity. In the LDE hutch, the small stages are each capable of housing up to three cells (*i.e.* three sets of service interfaces) and the medium stages each have one set of services for one cell. For large cells, these could be housed on the two large heavy-duty multi-axis goniometer stacks (Huber) mounted on a common heavy-duty linear drive. The small, medium and large stages all provide threaded breadboard mounting for sample cells. Type-R sample sets consist of 20-sample transmission plates, six-sample capillary frames, or bespoke, small, self-contained cells. These are stored in a dedicated sample rack (‘hotel’) and picked/placed onto a dedicated linear stage by a robotic arm (Yaskawa Motorman), prior to the stage translating into the beam. The sample hotel can house up to 12 Type-R cells. Use of a Schunk gripper for pick–place and locating the cells in both the stage and the hotel by the robot ensures positioning repeatability. The small, medium and Type-R linear drives as well as the detector frame are all mounted on a large granite table. The layout of the LDE hutch and the stages is shown by the drawing in Fig. 3 ▸(*a*), complemented by the CAD drawing viewed along the beam (Fig. 3 ▸ *b*).

### 2.3. Variable distance area detector   

**Figure 4:**
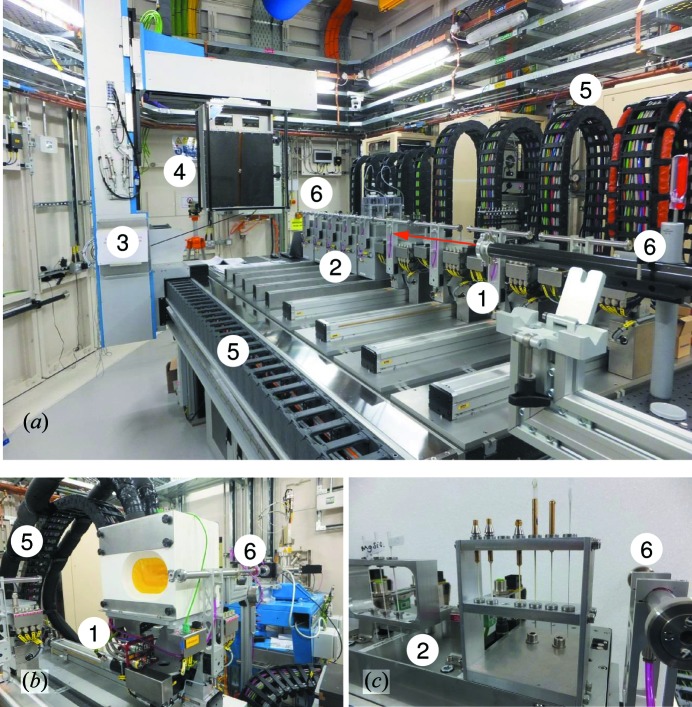
Photographs of the LDE facility: (*a*) key components on the large granite table, (*b*) the first medium stage (MS1) accommodating a low-temperature mineralization cell (room temperature, *i.e.* −30°C or 243 K) and (*c*) capillary samples on a small rack mounted onto the third small stage (SS3). The key features are labelled as (1) medium stages, (2) small stages, (3) large *XYZ* frame, (4) Pixium area detector, (5) energy (electrical) chains and (6) beam-pipes.

A Pixium digital area detector (RF4343, Thales) was chosen for the recording of two-dimensional powder diffraction patterns as it provides a large active area (430 × 430 mm) with a columnar crystalline CsI scintillator array for high-energy detection, in which X-rays are converted to visible light which is then detected by an underlying amorphous silicon photodiode array. Each pixel is 148 × 148 µm and there are 2880 × 2880 pixels within the active area. In addition, the Pixium has a point-spread function of ∼1–2 pixels and high dynamic range (16 bit, high detection quantum efficiency ∼65% with a rate of up to 30 frames per second). The processed signal is written to a two-dimensional file maintaining the 16 bit depth resolution. The characteristics and performance of the Pixium detector for synchrotron beamline use have been described by Daniels & Drakopoulos (2009 ▸) and Drakopoulos *et al.* (2015 ▸). The Pixium’s relatively light weight (∼25 kg) allows it to be mounted onto a large frame with *X*, *Y* and *Z* translation (Fig. 4 ▸ *a*), driven by encoded servo motors (see Table 4 ▸). This is crucial, since large movements (up to 3000 mm in the *Z* direction) with micrometre-level positioning repeatability are required to provide accurate, repeatable and programmable sample-to-detector distances for each experiment throughout its duration. The *X*–*Y* motion, as well as allowing centring of the detector, is also sufficiently large to allow the detector to be offset in either direction in order to sample more reciprocal space and improve resolution if required.

The commissioning photographs in Fig. 4 ▸(*a*) show the completed experimental hutch with small and medium sample stages and the detector installed on the large table. Also shown is the first medium stage (MS1) populated with the low-temperature mineralization or cold cell with five sample chambers (Fig. 4 ▸ *b*) and capillary samples on a small rack attached to a small stage (Fig. 4 ▸ *c*).

### 2.4. Machine protection, space management and safety features   

There are two beamstops to prevent the direct beam hitting the detector. The small primary beamstop is a tungsten cup mounted on the end of a long carbon fibre tube fixed to the detector’s *XYZ* stage. The primary beamstop is permanently in place, but its position is adjustable *via* a small *XY* motor. The larger secondary beamstop is a 25 mm lead disc mounted on a pneumatically driven retractable arm, and it is a protection device intended for use during commissioning and alignment. Detector motions are interlocked with the engagement of the secondary beamstop, preventing any accidental exposure of the ‘straight through’ intense beam running across the detector. Both beamstops have photodiode sensors to detect the beam for alignment and monitoring purposes.

Since the detector and sample stages need access to the same physical space at different times, a hardware and machine protection anti-collision scheme has been implemented at the underlying EPICS motion control level *via* a system of interlocks. This, for example, prevents a sample stage moving into the beam position if the detector is upstream of it. Similarly, the detector cannot move along the beam if a sample stage is already engaged. In addition to upper and lower travel limits, each stage on the large sample table has a parking switch such that the detector can only be driven past the stage if its parking switch is activated. Mounted around the detector, front and back, are light curtains to prevent cell parts, or other items that protrude beyond a stage’s edges, from hitting the detector surface. The detector frame also carries two cameras which point directly towards the sample cells and allow sample viewing and monitoring, while pan–tilt–zoom cameras strategically mounted on the hutch walls allow motions and positions to be checked visually from different angles during operation.

Since EH2 is constructed in series with EH1, it is necessary to install beam transfer pipes to transmit the beam from the EH1 sample position to the shutter connecting EH1 to EH2. This is achieved by parking the EH1 diffractometer detectors, removing the beamstop and installing two evacuated pipes that mount on pre-aligned stands that are located *via* threaded holes in the floor of EH1. The longer of the two pipes (∼3 m) is constructed from carbon fibre and both pipes are capped-off with Kapton. Installation takes a matter of minutes and, once in place, the diffractometer, sample table and robot in EH1 are locked into position. Similarly the EH1 beamstop, which is normally interlocked to the EH1 shutter, is set to override to allow the beam into EH2. With multiple multi-axis stages and provision of services to each cell, efficient cable management is important to allow easy maintenance and access, as well as providing good space usage within the hutch. All cables and services to the stages and detector are therefore gathered in energy chains as labelled in Fig. 4 ▸(*a*). These run under a raised floor to control racks (motion and detector) located outside the hutch *via* labyrinths, or a series of racks (cell controllers) located under the sample table and along one of the hutch walls.

## 3. Data collection procedure   

### 3.1. Acquisition features and routine   

LDE data acquisition is performed *via* the *Generic Data Acquisition* system (*GDA*, 2013 ▸), which is the standard data acquisition software across all Diamond beamlines. To collect whole powder patterns for a given sample or cell, the following procedure is adopted: (i) close the X-ray shutter and move any upstream cells from their parked positions to their ‘zero’ positions such that the short evacuated beam flight tubes attached to each stage line up sequentially to provide a low-background beam path up to the sample stage being measured; (ii) move the sample stage of interest to the predetermined in-beam position for its associated calibrant; (iii) move the detector to its predetermined mechanical sample–detector distance (with *x*–*y* centring/offset as required) and calibrate the detector dark current without the beam; (iv) open the hutch shutter and collect calibrant diffraction data for a predetermined exposure time; (v) using the certified lattice parameter automatically fit and integrate the calibrant data and refine pair-wise the wavelength and detector distance; (vi) move the sample stage to the predefined sample position(s) and collect data using predetermined exposure times; (vii) using the refined wavelength and detector distance, integrate the collected sample data and convert to intensity *versus* 2θ (or *q* as required). Steps (v) and (vii) are achieved *via* distributed cluster computing (initiated from *GDA*) and can be done while the next dataset is being collected if multiple collections on, for example, different regions within a given cell are required.

To implement this process in *GDA*, as well as the usual positioning commands, new data collection commands have been introduced which take the format lde *t* <*XXX*>, where *t* is the exposure time and <*XXX*> is a command option describing how the collected data should be processed. There are three options. (i) NDR, standing for ‘no data reduction’, tells *GDA* to collect and return just the raw detector image and is generally used during commissioning and alignment. (ii) CAL tells *GDA* that the data being collected are from a calibrant material, a high-quality NIST powder standard, *e.g.* CeO_2_, SRM674b (face-centred cubic). The wavelength (λ) and detector distance (*D*) should therefore be refined using the certified lattice parameter. (iii) SAM tells *GDA* that the data are from a sample and that the detector image should be integrated and converted to intensity *versus* 2θ using the refined λ and *D* values. Because of variations in mechanical positioning and X-ray beam energy from week to week, at least one CAL dataset is collected for every experiment each week. All *GDA* commands can be scripted so that, once the detector, cell and sample positions have been determined during commissioning, all weekly data collections are automated *via* initiating their scripts, along with the gathering of additional parametric data relating to cell conditions such as temperature and humidity.

### 3.2. Setup and schedule   

Apart from the actual design of the facility and its hardware, there are other operational issues that need to be addressed. It is impractical to either expect or require multiple user groups to attend site every week for months or years to collect data; and it is largely unnecessary. However, for this to be so, it is necessary not only for the hardware components to be automation compatible but for the whole LDE system itself to be compatible with high levels of automation from the outset. In standard user experiments at Diamond each user group operates the beamline *via* a separate instance of the *GDA* client, which accesses only their data storage area, with permissions determined by their user identification. Thus an LDE-specific client version of *GDA* has been developed to control the whole LDE facility from a single instance, capable of writing data to the correct storage areas. The operational model is therefore that, for each approved LDE, the user group will be on-site for no more than a few days to set up and commission the experiment. Once the experimental run starts, data collection for all operational LDEs is then done automatically and periodically, with the specific scheduling being preset *via* the beamline staff.

## 4. Commissioning results   

### 4.1. Measurement of powder diffraction standard   

**Figure 5:**
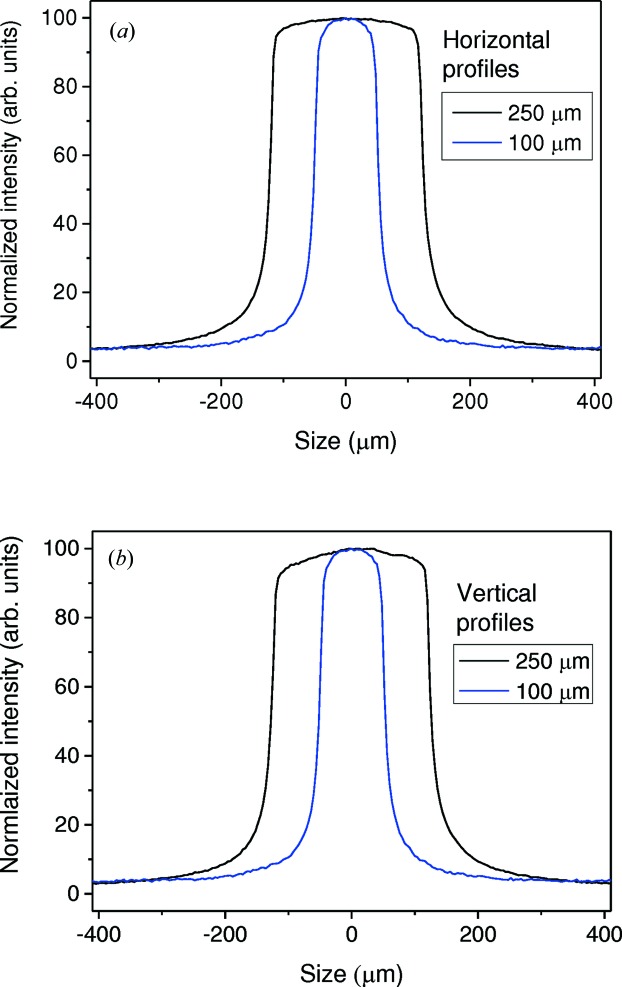
The profiles of the incident X-ray beam (*E* = 25 keV) measured in EH2: (*a*) horizontal and (*b*) vertical using a beam of 100 × 100 and 250 × 250 µm.

**Figure 6:**
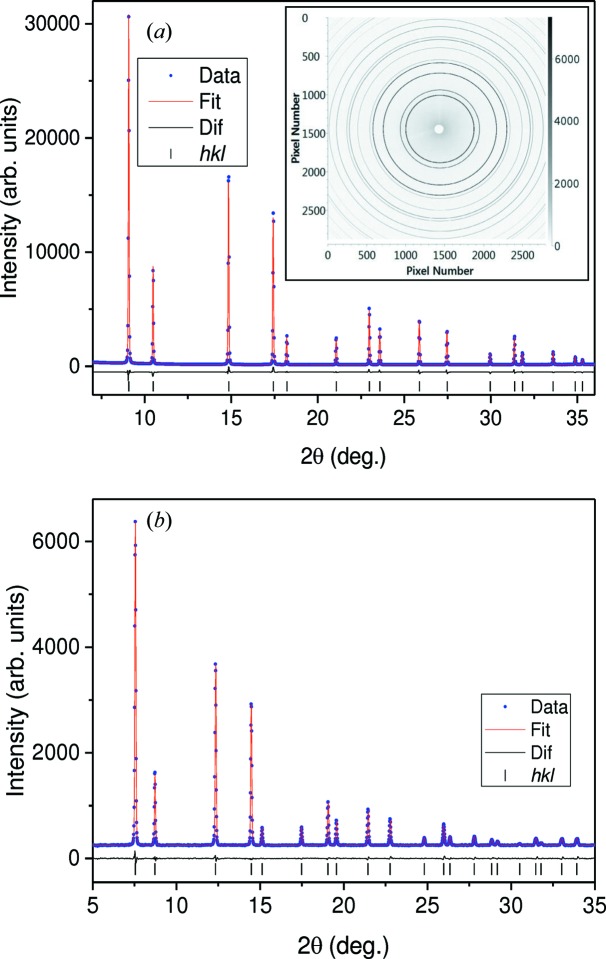
(*a*) SXPD pattern of CeO_2_ powder standard and Rietveld refinement (main plot) produced from the two-dimensional pattern (inset). The measurement was performed using a beam of 25 keV [λ = 0.49466 (1) Å] and 60 s exposure time. (*b*) SXPD pattern of CeO_2_ powder standard and the refinement obtained using a beam of *E* = 30 keV [λ = 0.41100 (5) Å] and 300 s exposure time.

The characteristics of the I11 incident beam have been extensively described previously (Thompson *et al.*, 2009 ▸) and we will only consider here the uniformity of the beam profile at the LDE operational energy of 25 keV. To produce high-quality data with good resolution, transmission geometry requires the use of a small beam size, typically 100–400 µm. Fig. 5 ▸ shows vertical and horizontal profiles measured for 100 × 100 and 250 × 250 µm beams. The well defined beam profiles (top-hat) have enabled the collection of high-quality data as demonstrated with the results presented here. For initial commissioning measurements a small disc (0.5 mm thick and 6 mm diameter) of SRM674b CeO_2_ was prepared by filling the central hole of a metal washer with the standard reference powder and sealing either side with 25 µm Kapton foil. Transmission powder diffraction patterns were obtained using a beam size of ∼400 × 400 µm, an electron beam current of 300 mA and a fixed (encoded) sample–detector distance of 400 mm. Fig. 6 ▸(*a*) shows a two-dimensional (inset) pattern obtained with 60 s exposure time and the reduced one-dimensional pattern (main plot) produced using the *Data Analysis Workbench* (*DAWN*; Basham *et al.*, 2015 ▸). The reduction process is an automatic feature which has been recently added to *DAWN* (Filik *et al.*, 2017 ▸). The reduced CeO_2_ data were fitted with the standard crystal structure of the oxide using the Rietveld method in *TOPAS* (Coelho, 2007 ▸). A good fit was obtained by fixing the fractional atomic coordinates of Ce and O according to the space group symmetry (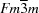), while the displacement parameters were successfully refined to be 1.38 (1) and 1.27 (4) Å^2^ for the two atoms, respectively. Owing to the detector integration of intensity and the data reduction process, counting statistics uncertainties are not available. Therefore, the usual refinement indictors (*R*_wp_ and *R*_exp_ factors) are not meaningful, but a low *R*_p_ = 2.47% was achieved by the fit. Good refinements can also be obtained from patterns collected using a beam of *E* = 30 keV (*e.g.* Fig. 6 ▸ *b*), but with a much longer exposure of 300 s, which was needed for good statistics owing to the substantial reduction of the beam intensity: a characteristic of the undulator source (Thompson *et al.*, 2009 ▸). This is the reason that the default operational energy is 25 keV for LDE measurements.

### 4.2. Repeatability and precision   

**Figure 7:**
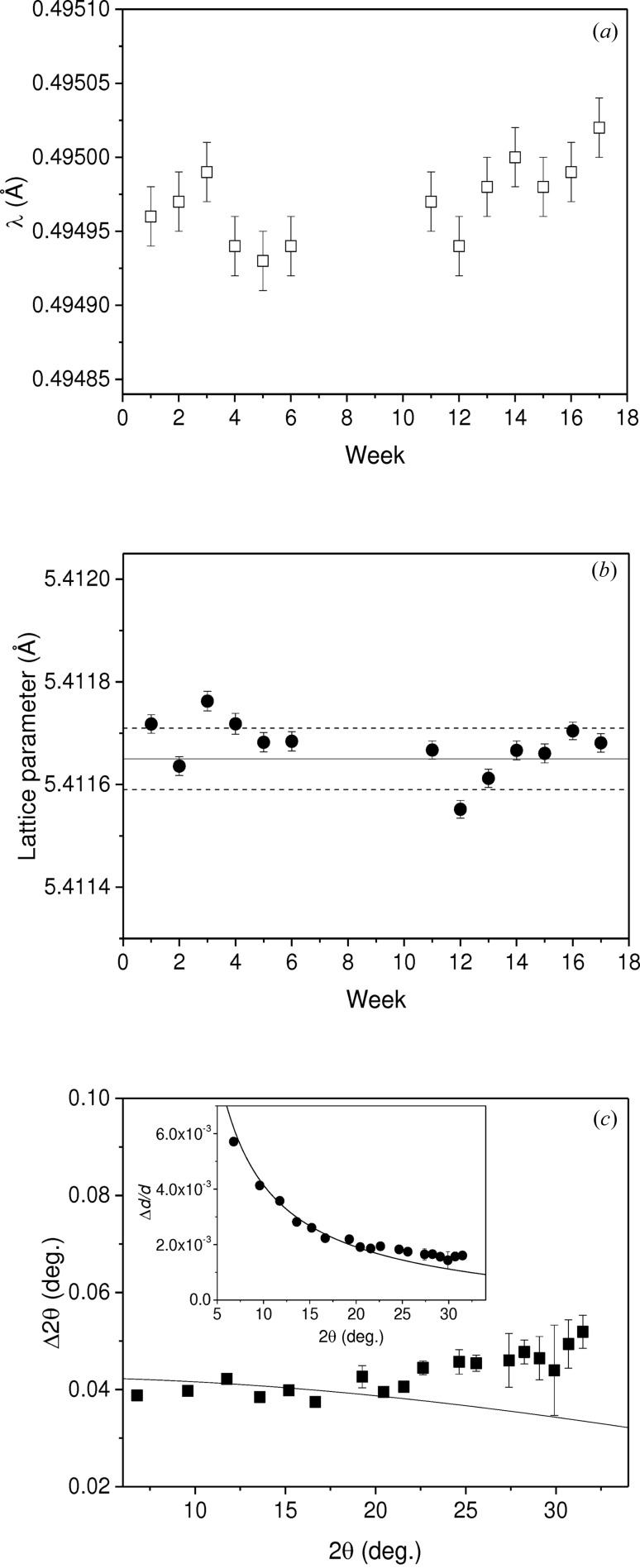
(*a*) Refined wavelength (λ) as a function of duration obtained from weekly measured CeO_2_ patterns after adjusting the monochromator to 25 keV each week. The absence of data between 7 and 10 weeks was due to a synchrotron machine shutdown. (*b*) The cubic unit-cell parameter as a function of duration, showing good reproducibility; the horizontal solid line is the certified value [*a*_0_ = 5.41165 (6) Å] and the dashed lines are the lower and upper limits of the error. (*c*) The instrumental resolution function represented by Δ2θ (main plot) and Δ*d*/*d* (inset) as a function of 2θ. The data were obtained from an LaB_6_ pattern (*D* = 400 mm, beam size = 200 × 200 µm and 25 keV beam).

It is important to know the repeatability and precision limits of the LDE instrument. A simple method of using another thin CeO_2_ standard (100 µm) fixed onto one of the small sample stages was devised. A diffraction pattern was measured every week for 17 weeks by the positioning of the area detector at the same distance to the sample. As the motions of the detector frame have precision encoders, the same *Z* distance (∼300 mm) can be achieved with high accuracy (a few micrometres). Initially, the determination of the distance [*D* = 308.49 (3) mm] was performed using the radiation of the Ag *K*-absorption edge (*E* = 25.514 keV) calibrated using a thin silver foil. As in operation, the weekly measurement was then carried out using a 25 keV beam. The refined results of the wavelength (λ) and lattice parameter (*a*) are presented in Figs. 7 ▸(*a*) and 7 ▸(*b*), respectively. The λ spread of ±5 × 10^−5^ Å is more than twice the individual refined error (2 × 10^−5^ Å). This is not entirely unexpected since the monochromator’s Bragg angle for the selection of the energy (25 keV) needs to be re-dialled every time the beam is transferred from EH1 to EH2, as the upstream hutch normally operates at 15 keV. Sometimes maintenance work during shutdowns could also affect the fine setting of the monochromator. However, the refined *a* parameters using the corresponding wavelengths show good reproducibility and high precision. With an average value of 5.41168 (2) Å and a spread of Δ*a* = ±0.00008 Å (Δ*a*/*a* = 1.5 × 10^−5^), the values are in excellent agreement with the certified lattice parameter, *a*_0_ = 5.41165 (6) Å, and most of them lie within the upper and lower error limits as shown in Fig. 7 ▸(*b*).

### 4.3. Resolution function   

As the beam produced by the I11 undulator is highly collimated, the resolution of the instrument is governed by the beam size, the sample properties (thickness and crystallinity) and the detector’s pixel size (§2.3). If one uses a high-quality thin sample and a sufficiently small beam, the resolution can be examined. Fig. 7 ▸(*c*) (main plot) shows the resolution function as Δ2θ *versus* 2θ, where the measured values are the full width at half-maxima of diffraction peaks extracted from the pattern of a 100 µm thin sample of LaB_6_ (SRM660b). This high-quality standard was chosen as it is known to have negligible contributions (particle size and strain) to the peak widths. The measurement was carried out with a sample–detector distance of 300 mm, beam size of 200 µm^2^ and beam energy of 25 keV. At low angles, the curve calculated using the model described by Hinrichsen *et al.* (2008 ▸) is in agreement with the experimental data. However, small differences between the observed and calculated curves are noticeable at high angles. The trend of deviation suggests that the discrepancy could be due to the calibrations of tilt and yaw of the panel detector. These angular parameters were obtained using the strong low-angle rings to calibrate the whole pattern. Therefore their accuracy could not allow precise location of the weak high-angle rings, which has a ‘smearing’ effect on the high-angle peaks resulting in the artificial broadenings. It should be pointed out that this is not an extensive modelling of the resolution function, but the results do demonstrate the achieved resolution (Δ2θ ≤ 0.05°) of the LDE instrument. The resolution function can also be presented as Δ*d*/*d versus* 2θ, as shown (Fig. 7 ▸ *c*, inset), illustrating that Δ*d*/*d* ≃ 2–6 × 10^−3^ can be achieved for the measured 2θ range. It should be pointed out that, for a real experiment, factors such as the beam size and the sample properties can also affect the data quality.

## 5. Results of first scientific experiments   

### 5.1. *In situ* aqueous formation of meridianiite (MgSO_4_·11H_2_O)   

**Figure 8:**
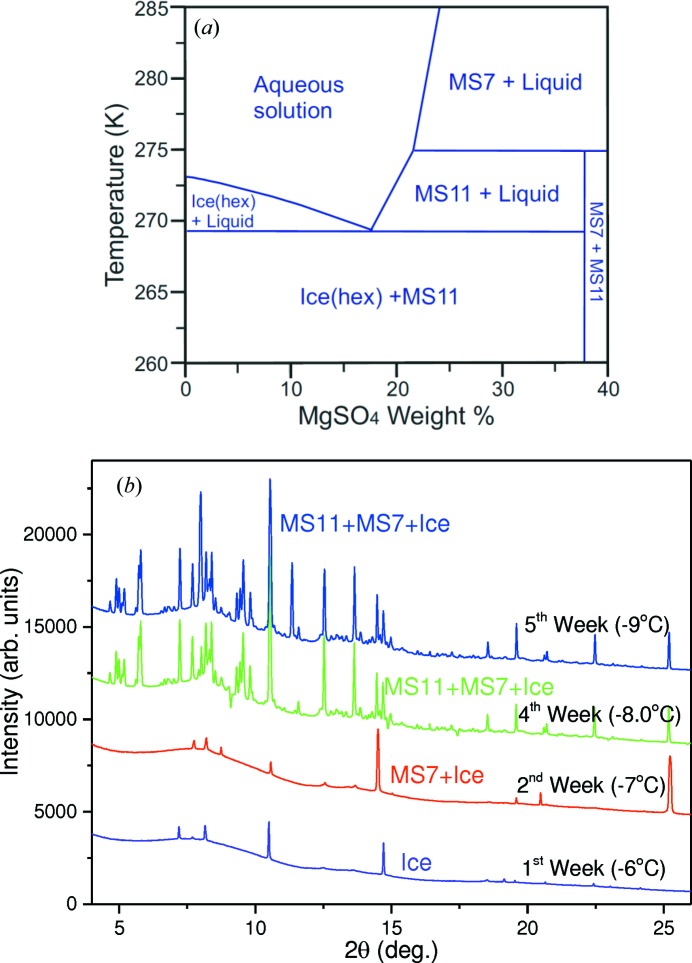
(*a*) Phase diagram of MgSO_4_·*n*H_2_O. (*b*) SXPD patterns showing the slow evolution of the MgSO_4_·*n*H_2_O system from ice to meridianiite in the cold cell [λ = 0.49419 (1) Å].

One of the central problems surrounding our understanding of Mars and whether it might once have harboured life is determining its hydro­logical history from present day observations. This requires knowing both the amount of water currently retained on the surface and the nature of the reservoir (*e.g.* hydrated minerals) holding it. Satellite orbital observations showed hydrated sulfate outcrops, several kilometres thick, in the walls of Valles Marineris (Bibring *et al.*, 2007 ▸). Although rare on Earth and limited to a few – mostly glacial and sea ice – occurrences, the undecahydrate sulfate mineral meridianiite (MgSO_4_·11H_2_O, hereafter MS11) is now believed to be widespread on Mars (Peterson & Wang, 2006 ▸; Squyres *et al.*, 2004 ▸). Indeed, the mineralogical name is derived from the Opportunity rover landing site in Meridiani Planum (a plain located 2° south of the Martian equator), where its presence was deduced *in situ* from rover observations. The MgSO_4_–H_2_O equilibrium phase diagram shown in Fig. 8 ▸(*a*) is adapted from the work by Hogenboom (1995 ▸), Peterson & Wang (2006 ▸) and Fortes *et al.* (2008 ▸) for the MgSO_4_·*n*H_2_O system. It suggests that MS11 is stable between −4 and 2°C (269–275 K and below), but forming it in the laboratory has thus far involved procedures not conducive to long-term *in situ* study by powder diffraction. Therefore the LDE instrument was used in an exploratory study of the formation of MS11.

#### 5.1.1. Sample preparation and LDE environment   

A transmission geometry cold cell (Fig. 4 ▸ *b*) was specifically designed, capable of housing multiple aqueous samples (×5), allowing differing environments to be simulated as part of a project aimed at studying long-term mineral precipitation in cold aqueous environments. For the work reported here an aqueous solution of ∼36 wt% concentration of MgSO_4_ was made by mixing 5 g of reagent grade MgSO_4_·7H_2_O with 24 g of 18 MΩ cm deionized water. This concentration was chosen as it would allow us to traverse three stability fields in the phase diagram (Fig. 8 ▸ *a*). The solution was loaded inside a chamber (5 mm deep × 25 mm diameter). The cell comprises a copper block body through which a refrigerant coolant is circulated. The small sample compartment has diamond windows for beam entrance and exit. The sample blocks are then mounted inside a thermally insulated body fitted with Kapton windows. The cell temperature was regulated *via* a Lauda chiller, with an antifreeze–water mix. For the work reported here a cooling ramp of −0.1°C d^−1^ was used, starting at a super-cooled temperature of −6°C (267 K) necessary to form water ice in the sample solution. SXPD patterns were collected every week for 2 months. During the experiment the cell was scanned vertically through the beam in order to provide representative sampling and to identify the positions of the brine-rich regions. It is within these regions that low-temperature mineral precipitation is expected to occur (Butler & Kennedy, 2015 ▸).

#### 5.1.2. Mineralization results   

The SXPD patterns in Fig. 8 ▸(*b*) show the evolution of precipitated phases within the cold cell at different times as the temperature is reduced. Following the formation of pure-phase hexagonal ice, the first precipitate phase was detected after 2 weeks and was identified as epsomite (MgSO_4_·7H_2_O, denoted as MS7), as predicted by the phase diagram (Fig. 8 ▸ *a*). By week 4, MS11 is observed, again as predicted by the phase diagram. The results of a Pawley fit using *TOPAS* to the pattern at −10°C (263 K) are listed in Table 5 ▸. The fits used a triclinic cell with space group *P*1 (*Z* = 2) and the initial lattice parameters reported by Fortes *et al.* (2008 ▸) from neutron data collected at 250 K as part of an investigation of the thermal expansion of MS11 from 4 to 250 K (−269 to −23°C). Their work also modelled the expansion behaviour using a third-order polynomial function. The expansion model was used to calculate the lattice parameters at −10°C (263 K) as recorded by the cell thermocouple. The results are listed in Table 5 ▸ for comparison. Given the limitations and limited accuracy of the model and that the fitting range used by Fortes *et al.* (2008 ▸) only goes up to 250 K (−23°C), the agreement of the calculated parameters is reasonably good. This shows that the temperatures being achieved by the cell are well represented by the thermocouple, confirming the effective design for this type of subzero temperature (°C) phase formation study.

The presence of MgSO_4_ on Mars probably arose from the reaction of basaltic material with sulfuric acid of volcanic origin and subsequent evaporation (Tosca *et al.*, 2005 ▸). Epsomite (MS7) when in contact with a saturated solution is unstable below ∼1.85°C (275 K), transforming to MS11. The commissioning results from the cold cell show the development of MS11 occurring somewhere between −7 and −8°C (266 and 265 K, *i.e.* 1–2°C below the super-cooled temperature required to initially form water ice) and over a period of up to a week, suggesting a kinetic contribution to the transformation process. The relatively quick uptake of water by the MS11 phase as observed here could explain why long-standing bodies of water are not observed on the planet. The results of this long-term study will be reported in detail in due course.

### 5.2. Long-term structural stability of gas capture material   

#### 5.2.1. Metal–organic framework NOTT-300   

Porous metal–organic framework (MOF) complexes show great promise for gas storage and separation owing to their high surface area and tunable functional pore environment (*e.g.* Davis, 2002 ▸; Li *et al.*, 2009 ▸). Within the field of gas capture, there is particular emphasis on optimizing the interactions between the MOF hosts and the adsorbed gas molecules, leading to the discovery of new functional materials with properties better suited to selective gas capture. Indeed, *in situ* experiments on I11 using the beamline’s gas delivery system have led to a number of high-profile publications in this field (*e.g.* Yang, Sun *et al.*, 2012 ▸; Chen *et al.*, 2014 ▸; Little *et al.*, 2015 ▸). One such framework complex is NOTT-300, which has been shown to have a high selectivity and uptake capacity to store harmful gases such as CO_2_ and SO_2_ (Yang, Lin *et al.*, 2012 ▸), and to separate C_2_ hydro­carbons (Yang *et al.*, 2015 ▸).

The microcrystalline white powder [Al_2_(OH)_2_(C_16_H_6_O_8_)]·6H_2_O (NOTT-300 solvate) was prepared using a previously published method (Yang, Lin *et al.*, 2012 ▸). The framework of the material consists of [AlO_4_(OH)_2_] chains bridged by biphenyl-3,3′,5,5′-tetracarboxylate ligands, crystallized in a body-centred tetragonal structure described by a space group *I*4_1_22 with *a* = *b* = 14.8296, *c* = 11.7732 Å and *Z* = 4. In the unit cell there are two channels running along the *c* axis with hydroxyl groups protruding into them along the 4_1_ screw axis, resulting in free hydroxyl groups oriented in four different directions along the pore. The diameter of the channels, taking into account the van der Waals radii of the surface atoms, is ∼6.5 × 6.5 Å. After desolvation the vacant channels can therefore be used to capture gas molecules. *In situ* SXPD studies of this material dosed with 1 bar CO_2_ or SO_2_ (1 bar = 100 kPa) confirmed the presence of O=*X*=O(δ^−^)⋯H(δ^+^)—O (*X* = C, S) hydrogen bonds, which are reinforced by weak supramolecular interactions with C—H atoms on the aromatic rings of the framework. These results explain the framework’s high selectivity of CO_2_ and SO_2_ over N_2_ and other small-molecule gases.

With a high surface area (1370 m^2^ g^−1^) and pore volume (0.433 cm^3^ g^−1^), NOTT-300 could be an excellent material for the application of new capture systems for CO_2_ and SO_2_. To realize its full potential, the long-term ability of gas capture and retention of the framework structure must be investigated, in particular when the material is loaded with a corrosive gas. The LDE facility is ideally suited to study the structural stability when the material is adsorbed with SO_2_ for several months as described in the next section.

#### 5.2.2. Gas loading of sample   

A powder sample of NOTT-300 was loaded in a gas capillary cell (Parker *et al.*, 2012 ▸) which was attached to a vacuum line. The sample was desolvated by heating up to 150°C (423 K), dosed with 1 bar of SO_2_ at room temperature and then transferred to the experimental hutch after the capillary was sealed. Fig. 4 ▸(*c*) shows a number of capillary samples of NOTT-300 loaded with different gases, including the one with SO_2_. A capillary loaded with CeO_2_ (SRM674b) was also used for instrumental calibrations. SXPD patterns were collected at three different positions of the sample using an incident X-ray beam of 25 keV.

#### 5.2.3. Structural results   

**Figure 9:**
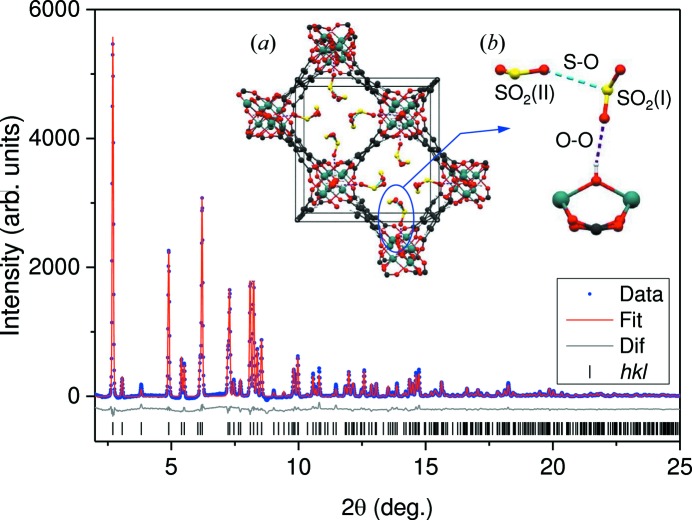
SXDP pattern of NOTT-300·4SO_2_: [Al_2_(OH)_2_(C_16_H_6_O_8_)](SO_2_)_4_ [λ = 0.49481 (2) Å] and Rietveld refinement results (main plot) with a refinement indication of *R*_p_ = 1.01%. (*a*) The crystal structure with adsorbed SO_2_ viewed along the *c* axis; atoms are shown as blue (Al), red (O), black (C), yellow (S) and white (H). (*b*) One of the four molecular arrangements in the channel, showing the locations of the SO_2_(I) and SO_2_(II) sites, and the S—O and O—O bonds, viewed along the *a* axis.

**Figure 10:**
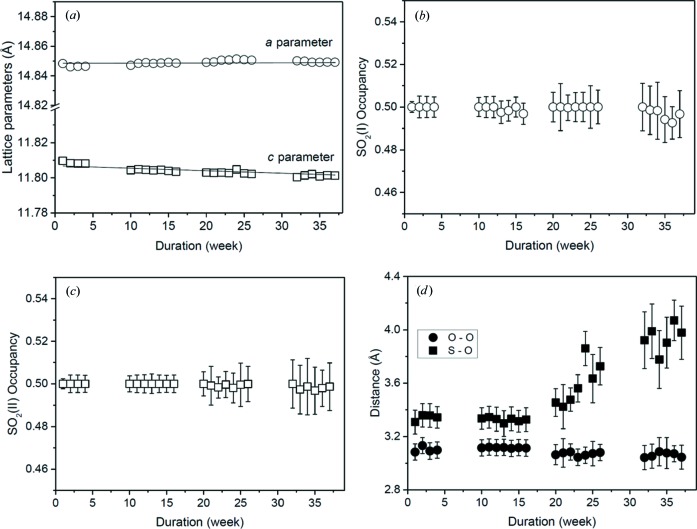
Lattice parameters of NOTT-300·4SO_2_ as a function of duration (*a*), site occupancies of SO_2_(I) (*b*) and SO_2_(II) (*c*), and S—O and O—O bond distances (*d*). The gaps without data are the shutdown periods of the synchrotron machine.

Using the structural model described in previous work (Yang, Lin *et al.*, 2012 ▸), Rietveld refinements of the LDE–SXPD data were performed in batch mode in *TOPAS* (Coelho, 2007 ▸). As an example, Fig. 9 ▸ (main plot) shows the data obtained at the beginning of the experiment and refinements including the occupation of SO_2_ molecules in the channel of the framework (Fig. 9 ▸ *a*). Two SO_2_ sites were found – SO_2_(I) is bound to the framework’s hydroxyl group *via* a hydrogen bond to the free hydroxyl groups on the pore surface, and SO_2_(II) is bound to SO_2_(I) *via* a dipole–dipole interaction, as shown in the molecular environment of Fig. 9 ▸(*b*). As SXPD patterns were measured for months, the unit-cell constants and other particular structural parameters can be used to monitor the stability of the MOF over this period. The results for the *a* and *c* parameters are presented in Fig. 10 ▸(*a*), showing some deviations in the data points since the refinement errors are quite small (Δ*a* ≃ ±0.0001 Å and Δ*c* ≃ ±0.0004 Å). This issue was due to our poor instrumental calibration procedure at the commissioning stage. Nevertheless, with averaged values of *a* = 14.8489 (1) Å and *c* = 11.8032 (4) Å, all the data are within Δ*a*/*a* = Δ*c*/*c* = 10^−4^–10^−5^. Interestingly, the linear fits show that the *a* parameter has changed little, while the *c* parameter is decreasing at a slow rate of 1.4 (2) × 10^−4^ Å per week.

The data analysis also provided structural atomic parameters and bond lengths. As for structural stability, it is important to closely examine the SO_2_ occupancies (Figs. 10 ▸ *b* and 10 ▸ *c*) and O—O bond distances between the two SO_2_ (I) and (II) molecules and from the hydroxyl oxygen to SO_2_(I) [the bonding configuration is shown in Fig. 10 ▸(*b*)] as a function of duration (Fig. 10 ▸ *d*). It is clear that there are no changes in the occupancies of each SO_2_ molecule, and only a small fluctuation in the position of SO_2_(I) relative to the framework’s hydroxyl group. The distance associated with O=S=O(II)⋯S(=O)_2_(I), however, begins to gradually increase after 20 weeks. This is due to the orientation of SO_2_(II) changing slightly, rather than any degradation of the framework. This has been shown by the retention of the high crystallinity, as confirmed by the diffraction data over 37 weeks. As the results here show, NOTT-300 appears to be a highly stable gas capture material. The work reported on here represents the preliminary proof of principle stage, whereupon a dedicated multi-sample LDE cell will now be designed that will allow for the *in situ* gas loading of a wide range of crystalline porous materials.

## 6. Conclusion   

We have successfully constructed and commissioned the world’s first dedicated facility for long-duration experiments. With specific design features as described, it has the capacity to accommodate up to 20 LDEs running in parallel to maximize the available beamtime. Although not an ultra-high-resolution instrument, the facility is capable of detecting phase evolution and detailed structural changes within the limits of its resolution (Δ*d*/*d* ≃ 10^−3^ and Δ*a*/*a* ≃ 10^−5^). This is well suited for many applied systems or functional materials of interest, such as the two scientific cases presented in this paper. The facility already has six LDEs running which are programmed for weekly data collection, ranging from long-duration degradation of nano-FePO_4_ cathodes in Li-ion batteries, to slow geochemical processes in cold aqueous environments, to long-term humidity effects on pharmaceuticals. Recently, five more experiments have been approved and commissioned. The emergence of new science from the ongoing experiments is expected soon, as demonstrated by the results from the first scientific commissioning experiments detailed above.

## Figures and Tables

**Table 1:** Beamline LDE components and key specifications

Photon source	In vacuum undulator
Energy range (wavelength)	*E* = 20–30 keV (0.41–0.62 Å), tunable
	*E* = 25 keV (default setting)
Optical elements	(*a*) Si(111) double crystal monochromator (Δ*E*/*E* = 10^−4^)
	(*b*) Harmonic rejection mirrors
	(*c*) Collimation slits
X-ray flux at sample at beam current = 100 mA	10^11^–10^12^ photons s^−1^ mm^−2^ (0.01%bw)^−1^ at 25 keV

Detection system (transmission geometry)	Large pixellated area detector: active area = 430 × 430 mm
Data acquisition speed	Seconds to minutes per pattern
Resolution	Δ2θ ≤ 0.05° (Δ*d*/*d* ≃ 10^−3^–10^−4^, for nominal 400 mm detector–sample distance)
Beam size at sample	100 × 100 to 500 × 500 µm

**Table 2:** The cell classification, services and other information

Class	Dimensions† (mm)	Weight (kg)	Services available	Platform/stage
1. Small cell	≤ 100 × 100 × 50	≤ 2	Electrical only	Small stage (SS)
2. Medium cell	≥ 100 × 100 × 50	≥ 2	Yes	Medium stage (MS)
	≤ 300 × 300 × 150	≤ 5		
3. Large cell	≥ 300 × 300 × 150	≥ 5	Yes	Large stage (LS)
	≤ 500 × 500 × 300	≤ 50		
4. Robotic	≤ 100 × 100 × 50	≤ 2	No (robotic)	Type-R
5. Special cell	–	–	–	Type-T

† Length × width × height.

**Table 3:** Experimental stages, specifications and locations

Platform/stage	Motion axis† (range)	Repeatability (resolution)	Max. load	No. of stages	Location
Small (SS)	*X* (800 mm)	±5 (1) µm	6 kg	5	Large table
Medium (MS)	*Y* (±50 mm)				
Robot (RS)	Speed = 5 mm s^−1^				

Large (LS)	*X*, *Z* (±50 mm)	±5 (5) µm	100 kg	2	Goniometer table
	Y (±25 mm)	±2.5 (2.5) µm			
	Arcs (×2) (±15°)	±1 (1) × 10^−6^°			
	φ rotation (−5 to 180°)	±1 (1) × 10^−6^°			

Type-R	Robotic	–	3 kg	1	See Fig. 3 ▸(*a*)

Type-T	Static	–	500 kg	1	See Fig. 3 ▸(*a*)

† *X* = lateral movement (across the beam), *Y* = vertical, *Z* = along the beam.

**Table 4:** Technical specification of the large *XYZ* frame

Axis	Range (mm)	Repeatability (resolution) (µm)	Speed (mm s^−1^)	Max. load (kg)
*X* translation (across)	415	±10 (1)	300	80
*Y* translation (vertical)	420	±10 (1)	150	80
*Z* translation (along beam)	3000	±8 (1)	76	80

**Table 5:** MS11 lattice parameters of triclinic cell refined from data collected at −10°C (263 K) compared with calculated values at the same temperature

	*a* (Å)	*b* (Å)	*c* (Å)	α (°)	β (°)	γ (°)
Calculated	6.7535	6.8181	17.2897	88.1466	89.4491	62.7041
Measured	6.776 (7)	6.798 (5)	17.29 (2)	88.17 (8)	89.70 (6)	62.57 (9)
